# A review of rigid point cloud registration based on deep learning

**DOI:** 10.3389/fnbot.2023.1281332

**Published:** 2024-01-04

**Authors:** Lei Chen, Changzhou Feng, Yunpeng Ma, Yikai Zhao, Chaorong Wang

**Affiliations:** School of Information Engineering, Tianjin University of Commerce, Tianjin, China

**Keywords:** point cloud registration, deep learning, partial overlap, network acceleration, neural networks

## Abstract

With the development of 3D scanning devices, point cloud registration is gradually being applied in various fields. Traditional point cloud registration methods face challenges in noise, low overlap, uneven density, and large data scale, which limits the further application of point cloud registration in actual scenes. With the above deficiency, point cloud registration methods based on deep learning technology gradually emerged. This review summarizes the point cloud registration technology based on deep learning. Firstly, point cloud registration based on deep learning can be categorized into two types: complete overlap point cloud registration and partially overlapping point cloud registration. And the characteristics of the two kinds of methods are classified and summarized in detail. The characteristics of the partially overlapping point cloud registration method are introduced and compared with the completely overlapping method to provide further research insight. Secondly, the review delves into network performance improvement summarizes how to accelerate the point cloud registration method of deep learning from the hardware and software. Then, this review discusses point cloud registration applications in various domains. Finally, this review summarizes and outlooks the current challenges and future research directions of deep learning-based point cloud registration.

## 1 Introduction

With the rapid development of modern information technology and scanning equipment, point cloud data (PCD) has become the primary data format to represent the 3D world. Point cloud has numerous applications in different areas, including robotics (Fioraio and Konolige, 2011; Pomerleau et al., 2015), biomedical imaging (Min et al., 2018), road and architectural mapping (Chen et al., 2013), urban modeling (Chen et al., 2020), autonomous driving (Nagy and Benedek, 2018), and augmented reality (Tâche et al., 2009; Liu et al., 2020). 3D point cloud registration is a crucial and complex issue in point cloud data processing. In general, point cloud registration takes a pair of unregistered point clouds as input: source point cloud and target point cloud. The objective of point cloud registration is to determine a rigid transformation that aligning two point clouds Through registration, we can combine the point cloud data in the same scene or partial scanning the target data to generate a complete 3D point cloud.

**Figure 1:**
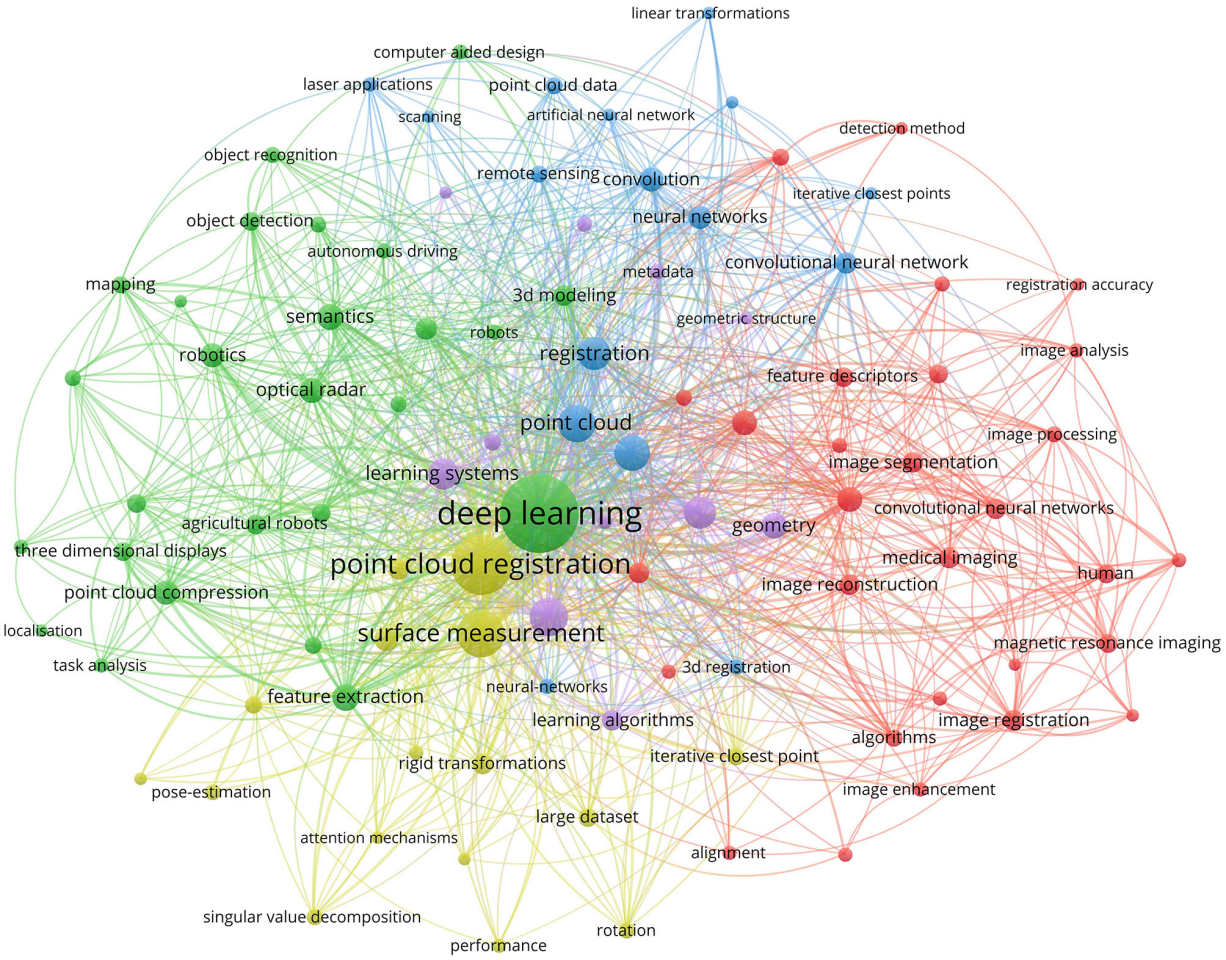
Visualization analysis of deep learning-based point cloud registration and related fields. The data are from the database of Web of Science.

Traditional point cloud registration methods such as ICP (iterative closest point) (Besl and McKay, 1992), NDT (normal transform) (Biber and Straßer, 2003), 4PCS (4-points congruent) (Aiger et al., 2008), and Random Sample Consensus (RANSAC) (Fischler and Bolles, 1981) etc., have found extensive applications in diverse fields. However, the majority of these methods are sensitive to noise, outliers, low overlap, and initial pose. The widespread adoption of deep learning in various domains, including computer vision, has garnered significant attention from researchers. Through consulting the current literature on point cloud registration based on deep learning, this article uses VOSviewer (A literature visualization analysis software) to do a visual analysis, and the data comes from the Web of Science (WOS) database. As shown in Figure 1, the size of each node represents the frequency of occurrence of the corresponding evidence in the relevant literature. It can be seen from the figure that point cloud registration based on deep learning involves many disciplines such as image segmentation, neural network, etc. Recent research focuses on feature extraction, attitude estimation, surface measurement, etc.

This article aims to provide a comprehensive and novel review of deep learning-based point cloud registration. To the best of my knowledge, only a few review articles have analyzed and summarized the point cloud registration of deep learning. Huang et al. (2021b) divided point cloud registration into homologous registration and cross-source registration, and discussed traditional methods and deep learning-based methods. Zhang et al. (2020) distinguished the popular deep learning-based methods according to correspondence and non-correspondence and analyzed of the principles of the various methods. Bello et al. (2020) analyzed and summarized the existing deep learning-based methods based on raw point clouds and also introduce the popular 3D point cloud benchmark datasets.

Compared with previous articles, the main contribution of this article is as follows: Firstly, the deep learning-based point cloud registration is divided into complete overlapping point cloud registration methods and partial overlapping point cloud registration methods. Complete overlap registration is valuable in scenarios where the captured point clouds cover the same scene from different perspectives or at different times. It enables the generation of a comprehensive and detailed representation of the scene by merging multiple scans. Incomplete overlap registration is particularly relevant when dealing with challenging scenarios such as fragmented or sparse point clouds, occlusions, or when there are limitations in data acquisition. Partial overlap registration allows for the integration of different data sources or incomplete scans to create a more complete and informative representation of the scene. This article focuses on the advantages and disadvantages of different methods, the development trend, and data processing methods. Secondly, the acceleration of registration methods is introduced in detail from the aspects of software and hardware to adapt to the increasingly large-scale point cloud data. And then, the application of point cloud registration based on deep learning in various areas is also summarized and commented in detail. Finally, we discuss directions of future research.

The remaining portion of the article is organized as follows: In Section 2, the basic principles of point cloud registration are briefly described, and the development of registration methods is introduced. In Section 3, the point cloud registration technology for complete overlap is discussed comprehensively. In Section 4, the state-of-the-art approach to partial overlap is discussed. Section 5 discusses how to accelerate the point cloud registration method based on deep learning from the hardware and software, so as to adapt to more and more large-scale point cloud data. In Section 6, the practical application and future directions of point cloud registration and a number of deep learning in various areas are summarized and commented. And then section 7 summarizes the work of this review, discusses the vigorous development of point cloud registration technology based on the deep learning, and makes specific prospects for future research. Finally, Section 8 gives a brief conclusion of the work in this article.

## 2 Preliminary knowledges

It is often challenging to obtain a complete point cloud of a target object simultaneously using 3D scanning devices and similar equipment. It is frequently required to scan from multiple angles to get the complete point cloud of the target object. And these point clouds may not be in the same coordinate system, there is a spatial rotation and translation relationship.

Given two point clouds: source point cloud $X=\left\{x_{i}\in R^{3}\right\},i=1,2,...,N$ and target point cloud $Y=\left\{y_{j}\in R^{3}\right\},j=1,2,...,M$. Where, *N* and *M* respectively represent the number of points in the source point cloud and the target point. Point cloud registration aims to solve the relative transformation from source point cloud *X* to target point cloud *Y* in the coordinate system, including the rotation matrix *R*∈*SO* and the translation vector *t*∈*R*^3^. Where, *SO* is the three-dimensional rotation group. Point cloud registration can be regarded as the least mean square error problem:


(1) $$\begin{array}{l} \underset{R,t}{\arg\min}\frac{1}{N}\overset{N}{\underset{i=1}{\sum }}{||Rx_{i}+t-y_{m}||}^{2} \end{array}$$

where, *y*_*m*_ represents the corresponding point of any point *x*_*i*_∈*X* in the source point cloud in the target point cloud. Equation (1) allows *R* and *t* to be solved by singular value decomposition (SVD). However, the corresponding point *y*_*m*_ is usually unknown, so before solving the transformation, the corresponding relationship between the starting point pairs needs to be established:


(2) $$\begin{array}{l} m=\underset{j\in \left\{1,...,M\right\}}{\arg\min}||Rx_{i}+t-y_{j}|| \end{array}$$

From the relationship between Equations (1), (2), we can see that the solution of Equation (2) depends on the known *R* and *t*, which is precisely the solution objective of Equation (1). Therefore, the conventional approaches such as ICP assumes initial *R* = *I* and *t* = 0. First, the nearest neighbor is used in Euclidean space to establish the correspondence *m*, then Equation (1) is used to solve the rigid transformation, and the above two processes are cycled until convergence. As a result, the method is sensitive to the initial pose and easily falls into local optimal and is difficult to continue optimization.

With the aim of enhancing the registration effectiveness of conventional algorithms, Gold et al. proposed a robust point-matching method (Gold et al., 1998), using annealing parameters to determine the minimum distance between points. The issue of ICP being susceptible to getting trapped in local optima is mitigated to some extent. Yang et al. (2013) proposed an iterative nearest point method (Globally Optimal ICP, Go-ICP) based on ICP. Through the alternate use of the branch definition method and ICP, the branch definition method is used to find a better solution when ICP falls into the local optimal, and obtain the better optimization results. However, this method improves the registration accuracy, but also significantly increases the registration time. To improve the running speed of the method and save the consumption time of Random Sample Con-sensus (RANSAC) (Fischler and Bolles, 1981), Zhou et al. (2016) proposed Fast Global Registration (FGR), using iterative optimization techniques to speed up the iterative process of the method.

Compared with traditional optimization methods, the point cloud registration method based on deep learning has significant advantages. It provides better robustness in dealing with challenges such as noise, low overlap, and large-scale data. In addition, it supports automation and end-to-end learning, eliminating the need for manual parameter tuning and feature engineering. Deep learning-based approaches can learn meaningful features directly from raw point cloud data (Li et al., 2020b; Spezialetti et al., 2020; Marcon et al., 2021), going beyond the limitations of manual features used in traditional approaches. In addition, deep learning models have better generalization capabilities, enabling them to adapt to a variety of scenarios (Zhao et al., 2019). Overall, deep learning-based point cloud registration methods provide more accurate, efficient, and adaptable solutions that drive advances in a variety of applications.

**Figure 2:**
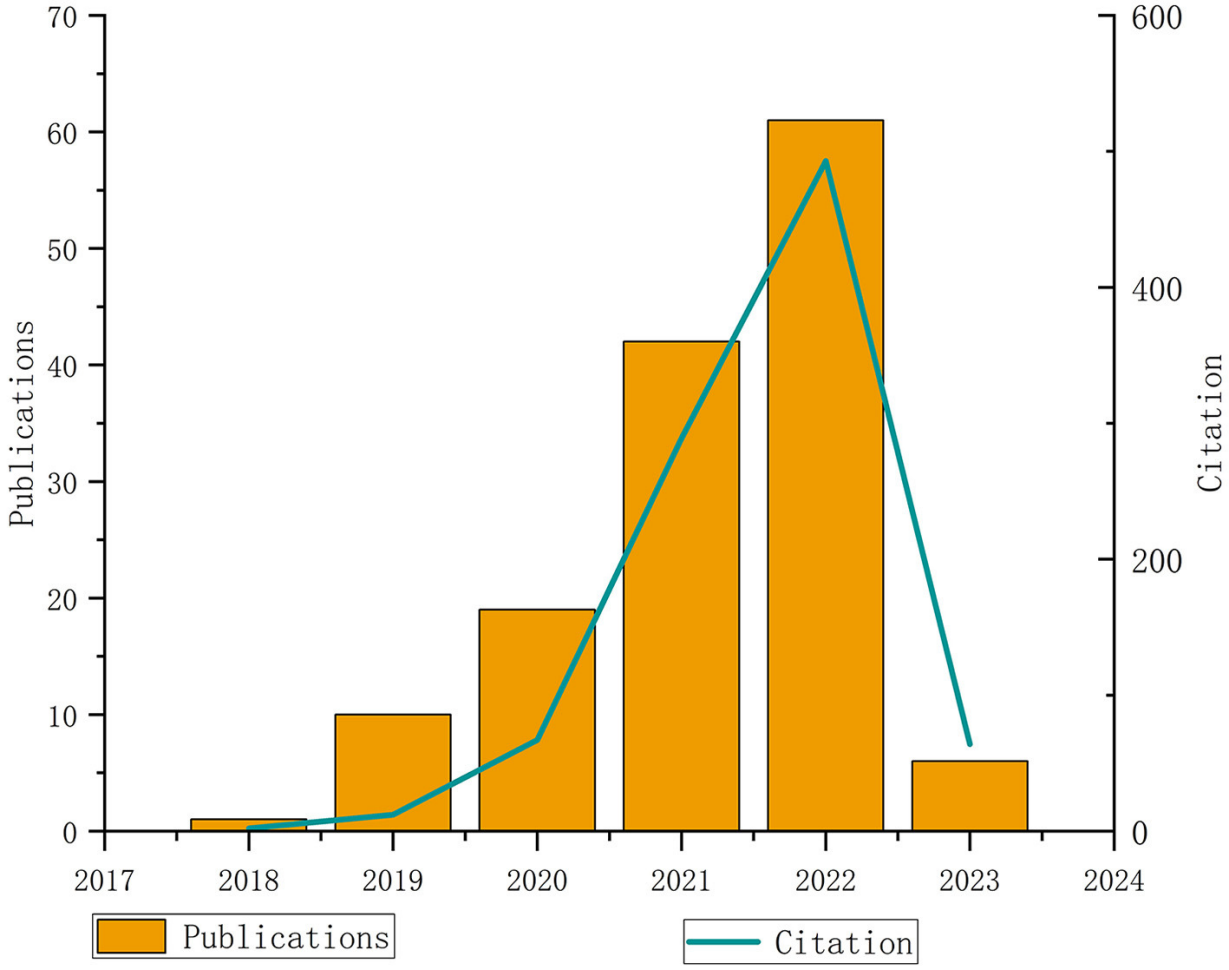
Analysis of publications and frequency of citations (Source: Web of science database).

In conclusion, the above conventional approaches need to be more robust to solve the local optimal problem, so they are only suitable for the rough registration process. At the same time, there needs to be more effective countermeasures for the partial overlapping point cloud registration. With the application of deep learning in the field of point cloud registration, a number of methods have emerged to solve the problem of low overlap point cloud registration. As shown in Figure 2, it can be seen that since 2016, the relevant literatures about deep learning point cloud registration have shown a blowout growth and are still increasing. According to the overlap of point clouds, considering that the point clouds will partially overlap in practical applications. This article divides the deep learning-based point cloud registration methods into complete overlapping point cloud registration and incomplete overlapping point cloud registration.

## 3 Complete overlap point cloud registration

In order to address the challenges posed by the intricate topology of point cloud surface, large scale of point clouds data and high robustness requirements, researchers have drawn inspiration from traditional methods and integrated deep learning techniques to decompose point cloud registration into several essential technologies. The most mainstream method is based on matching the relation of corresponding points and global feature-based methods.

**Figure 3:**
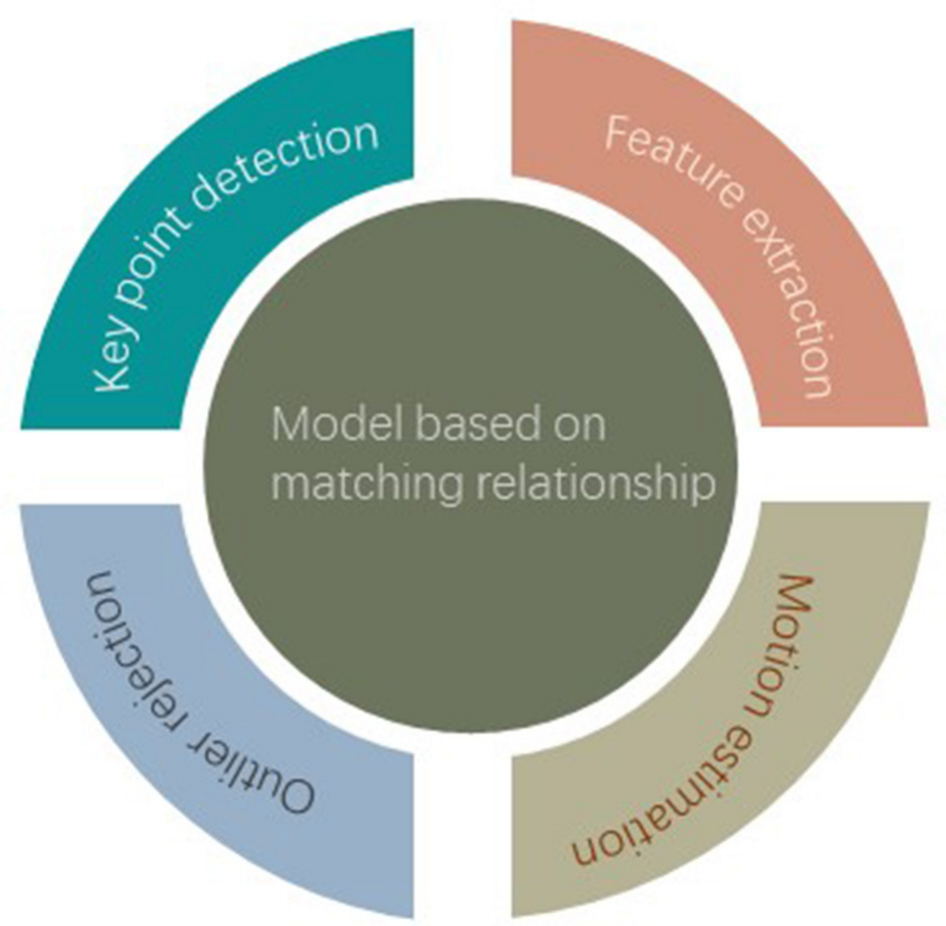
Model based on matching relationship.

Many researchers split point cloud registration on real data into multiple sub-problems are studied, as shown in Figure 3. In the synthesis data, most of the existing studies have adopted the end-to-end method. This section will discuss the complete overlapping point cloud registration technology based on deep learning. This article categorizes existing research on deep point cloud registration into two main approaches: correspondence-based methods and global feature-based methods. Furthermore, the end-to-end networks are also classified.

### 3.1 Model based on matching relationship

In order to facilitate comparison, this article classifies each method according to its main functions, feature extraction, key point detection, outlier rejection, and motion estimation.

#### 3.1.1 Feature extraction

The main idea of feature extraction method is to use deep learning to extract features to estimate accurate correspondence. A one-step optimization (SVD, RANSAC) can then be used to estimate the transformation without iterating between the corresponding estimate and the transformation estimate. Since point cloud contains abundant spatial geometry information, organizing point cloud reasonably and extracting more recognizable information is the most concerning in feature extraction. Qi et al. (2017) proposed PointNet, which was the first deep learn-based network model that extracted features directly from the input point cloud. The method applies deep learning to point clouds in a simple way and solves the disordered permutation problem with symmetric functions. The model generates descriptors for the global features of each point or the whole point cloud, and solves the problems of disorder, permutation invariance, and rotation invariance of the point cloud. At the same time, features are extracted from each point by multilayer perceptron (MLP), and the purpose of displacement invariance is achieved by symmetric function. Finally, T-net (trans-formation network) was used to predict the rigid transformation to satisfy the rotation invariance.

However, the PointNet ignores the relevance between points in the learning process, and the pooling operation is accompanied by a certain degree of information loss, which restricts the scene understanding ability of the network. In view of the above shortcomings, PointNet++ network [20] constructs a feature learning model of “sampling + neighborhood clustering.” PointNet ++ introduces a hierarchical structure consisting of the farthest sampling layer, the packet layer, and the PointNet layer to capture contextual information at different scales.

In general, PointNet and PointNet++, as a local feature extraction module, proves to be an effective learning structure, but it lacks the acquisition and learning of the relationship between point pairs, which limits the overall learning ability of the network. In order to solve the problem of limited learning ability of PointNet structures and extract more discernable information from unorganized point clouds, some researchers have proposed a feature extraction method based on convolution.

In order to solve the problem of limited learning ability of PointNet structures, different local region learning structures were designed based on deep convolutional networks. Wang et al. (2019) designed dynamic graph convolutional neural network DGCNN based on GNN. The EdgeConv structure is designed as the learning module of the local features of point cloud in the idea of fusion graph convolution. The neighborhood points are aggregated in the feature space, and the local features are learned through the graph convolution form. In DGCNN, the graph nodes will be dynamically updated at each layer of the network, which means that the nearest neighbors of a point will change layer by layer as the number of network layers increases. This feature makes EdgeConv very flexible and helps to obtain local topology information. But again, the increase of point nearest neighbor also brings additional K-nearest neighbor calculation loss. Zeng et al. (2017) proposed a data-driven method 3DMatch. Geometric descriptors of local regions were learned using 3D convolutional neural networks (3DConvNet) to establish correspondence between partial 3D point clouds. In order to optimize the descriptor based on 3DConvNet, a large amount of training data is required. For this purpose, corresponding tags in the existing RGB-D scene reconstruction are used to collect the training data. For real data registration, the two corresponding key features are usually registered on different scales. In order to obtain multi-scale features and avoid the loss of corresponding points caused by random sampling and downsampling, Socher et al. (2011) proposed a point cloud registration network based on multi-scale features, MSP-NET. In this method, Siamese multi-scale structure is used to downsample and upsample point clouds in layers to obtain multi-scale characteristics of key points. Meanwhile, the local similarity estimation module (LSEM) is used to locate the key points. In addition, in order to deal with the low overlap, the global estimation module (GSEM) is introduced to make the downsampling more concentrated in the overlap area. This module can find the corresponding points well with the two-sided outlier removal mechanism based on multi-scale features. This method can extract and utilize the features of multi-scale point clouds well and avoid the interference of outliers. However, when the central region of the point cloud to be registered is difficult to distinguish, the registration accuracy of this method is faster.

Full convolutional geometry learning in 2D data has been proven to be an effective method for feature extraction (Long et al., 2015). Based on this, Choy et al. (2019) have proposed fully convolutional geometric features (FCGF), which use sparse convolutions to replace the traditional 3D convolution. In this method, all the fully connected layers of the multi-layer perceptron are converted into a series of convolution layers with kernel 1*1*1, and negative mining is applied to the contrast and triple loss functions. The feature dimension of FCGF output is only 32 dimensions, which improves the operation efficiency of the method and can be applied to real scenarios. However, FCGF has the disadvantage of data sampling overfitting, resulting in poor generalization ability. Based on this, Horache et al. (2021) proposed multi-scale architecture and self-supervised fine-tuning (MS-SVConv) convolutional neural network. Methods A 3D sparse voxel convolutional network was used to compute the features on different scale point clouds, and then the features were fused by fully connected layers. The MS-SVConv inherits the advantages of FCGF's fast running speed, but also dramatically enhances the generalization capability. Thomas et al. (2019) proposed kernel point convolution (KPConv) in order to deal with the sparse and disordered structural characteristics of point clouds. KPConv, inspired by image-based convolution, uses a set of kernel points to define the region where the weight of each kernel is applied. The number of kernel points is unlimited. KPConv produces different offsets at each convolution location, which means that it can adjust the shape of the kernel according to different regions of the input cloud. However, because the combination weight is artificially set, the optimal result cannot be guaranteed. At the same time, in the face of different point clouds, different nuclear point Spaces need to be customized, limiting the network's generalization ability. Therefore, Xu et al. (2021b) proposed position adaptive convolution (PAConv). The coefficients of the weight matrix in the network are learned adaptively from the point position by Score-Net, and the convolution kernel is constructed by dynamically combining the basic weight matrix stored in the weight library. In practical applications, PAConv can replace MLP modules without changing the network structure and parameters, which makes the network more flexible.

To solve the unorganized problem of point clouds, researchers use grid (Yi et al., 2017) [22], voxel (Maturana and Scherer, 2015), and K-nearest neighbor to organize point clouds, so as to extract richer geometric features. Khoury et al. (2017) proposed an accurate and compact deep network of geometric features (CGF), which optimizes high-dimensional histograms to low-dimensional Euclidean Spaces. To get a series of more compact and accurate feature descriptors parameterized by dimension. Deng et al. (2018b) proposed PPFNet, the model can integrate local features and global features at different scales. Unlike voxel, the method makes full use of the sparsity of source point clouds and uses a new n-tuple loss function and architecture to inject global information into local descriptors naturally, improving the rotation invariance of features and robustness to noise. However, since the input features come from the point pair feature PPF (Rusu et al., 2008), it strongly depends on normal vector estimation. To improve the shortcomings of PPFNet, Deng et al. (2018a) further proposed PPF-FoldNet for unsupervised learning of 3D local descriptors in point clouds. In this network, the source point cloud and normal vector are not included in the coding, but the point cloud features are sent to the automatic encoder (AE) like FoldingNet (Hinton and Zemel, 1993; Yang et al., 2018; Liu et al., 2019a). After training, the set distance can be used to reconstruct the point pair features. This method does not need to sample more than three sets of point pairs from the pre-annotated data set for singular value decomposition, so the network training is more accessible and can still achieve good results under the condition of noise, but it is more sensitive to the density change of point cloud. Yew and Lee (2018) use Siamese neural network architecture to propose a weakly supervised form of 3DFeatNet. Methods 3D feature detectors and descriptors are learned from GPS/INS tagged 3D point clouds as a whole, and Siamese architecture (Varior et al., 2016) is used to learn to recognize whether a given point cloud is taken from the same location. Weights are learned through the introduced attention mechanism to measure the contribution of each input symbol to the triplet loss function. Finally, the network is trained by minimizing the triplet loss function.

Although features can be extracted directly from the point cloud using the Point-Net structure, this model restricts the further operation of the convolutional network. For this purpose, Gojcic et al. (2019) proposed 3DSmoothNet, which used voxelized smoothing density value (SDV) to match 3D point clouds with Siamese neural network deep learning architecture and complete convolutional layers. The full convolution layer computes the points of interest and aligns with the local reference frame (LRF) to achieve rotational invariance. The method can generate low-dimensional, highly descriptive features and intercommunicate these features between different sensors and indoor and outdoor scenes. This undoubtedly enhances the generalization ability of the network. At the same time, the low dimensional feature descriptors generated by the method (only 16 or 32 output dimensions) greatly speed up the corresponding search, which makes the realtime application of the method possible.

The local feature extraction method to ensure the input network's rotation invariance brings additional computing consumption. At the same time, it is sensitive to the initial pose of the source point cloud and noise anomalies. For this reason, researchers use convolution operation to make the model more efficient and obtain more significant local topological information, thus improving the visibility of features. The relevant methods in this section are summarized in Table 1 (Guo et al., 2021; Zhao et al., 2021; Yu et al., 2022b).

**Table 1:** Feature extraction methods.

**Model**	**Overview**	**Reference**
PointNet	First to use deep learning directly to extract features directly on a point cloud	Qi et al. (2017)
PointNet++	Uniform sampling with segmented local areas on top of PointNet	Qi et al. (2017)
DGCNN	Combining graph convolution with Edge-Conv for local feature extraction	Wang et al. (2019)
3DMatch	Learning local area geometric descriptors using 3DConvNet	Zeng et al. (2017)
FCGF	Replacing traditional 3D convolution with sparse convolution	Choy et al. (2019)
MS-SVConv	Using 3D sparse voxel convolution networks on different scales point clouds	Horache et al. (2021)
KPConv	Using kernel point convolution to define the weights of different regions	Thomas et al. (2019)
PAConv	Adaptive learning weight matrix coefficients	Xu et al. (2021a)
CGF	Mapping high-dimensional histograms to low-dimensional Euclidean spaces	Khoury et al. (2017)
PPFNet	Fusion of local and global features at different scales	Deng et al. (2018a)
PPF-FoldingNet	Adding the self-encoder AE from the Folding network to PPFNet	Deng et al. (2018b)
3DFeatNet	Learning point cloud features using Siamese neural networks	Yew and Lee (2018)
3DSmoothNet	Matching Siamese neural network with full convolutional layers by SDV	Gojcic et al. (2019)
MSPR-Net	Multiscale features and stratified sampling	Yu et al. (2022a)
PointTrans	The self-attention mechanism is applied to the point cloud	Zhao et al. (2021)
PCT	Improve point cloud Transformer with optimized offset-attention module	Guo et al. (2021)
Point-BERT	Using Bert to build a Transformer pre-training organization for point clouds	Yu et al. (2022b)

#### 3.1.2 Key point detection

Only more than three pairs of effective solution points are needed for point cloud registration to solve the rigid transformation. But random sampling of the point on the inevitable received noise, point cloud density impact. Therefore, the researchers developed a method designed to sample the point pairs that significantly impact the registration task.

Generally speaking, the detection of key points needs to predict the significance of points. Lu et al. (2020) proposed a key point detector and descriptor network (RSKDD-Net) based on random sampling. The model uses random sample to quickly collect key points in the scene and learn the features of local regional structure information. To solve the problem of information loss in random sampling, a new random expansion clustering strategy is used to enlarge the receiving field of each sampling point. The network contains a neighborhood point aggregation module based on an attention mechanism to dynamically sample key points by sensing neighborhood structure. Finally, the distance and point-to-point loss functions based on probability chamfer are used to supervise the training network. The network performs well in registration accuracy and is robust to point cloud noise and local sparse inequality. Ghorbani et al. (2022) proposed a Uniform and Competency standards-based 3D Keypoint Detection (UCKD). In this method, key points were first extracted by 3DSIFT or 3DISS detector, and different criteria were used to evaluate the quality of key points, and the final key points were selected according to the capability criteria. Then the octree structure is used to create a uniform spatial distribution in the point cloud, and the key points are extracted proportionally. Finally, the model uses orientation histogram (SHOT) descriptor to describe key points to complete registration. This method is robust for the registration of point clouds with appropriate distribution and low overlap. However, this method is only applicable to point clouds with uniform structure, and it is also a crude registration method, which needs to be combined with acceptable registration method to improve the registration accuracy. Bai et al. (2020) proposed D3feat by utilizing 3D complete convolutional network and combining with a brandnew learning mechanism that can intensively predict detection scores and descriptive features of each 3D point. To overcome the inherent density variation of 3D point clouds, the model evaluates the relationship between a point and its local neighborhood through the significance fraction of constant density. The key point score is calculated by combining the significance score of the point pair and the score at the end of the channel. Xu et al. (2021b) introduced the convolutional layer PAConv into the network and proposed a new point cloud registration network model PACNet (Ko et al., 2018) based on deep learning. The model can learn the weight coefficient according to the position relation between each point and its adjacent points, and adaptively construct the convolution kernel with the weight matrix. By integrating the local correlation and global information of key points, the information of local areas can be captured flexibly, which improves the understanding ability of the method for different scenarios. Chen et al. (2021) proposed a neural network VK-Net to discover a set of category-specific key points from a single point cloud in an unsupervised manner. VK-Net can generate semantically consistent and rotationally invariant key points between objects of the same category and different views. In general, the key point detection module requires additional structures to learn the location of virtual key points, resulting in many network parameters and computation. Therefore, it is only applicable to small-scale point cloud computing.

Usually, key point detection needs to be used together with feature extraction and other modules. Therefore, the key point detection module is embedded in many end-to-end registration methods. The relevant models including key point detection are summarized in Table 2.

**Table 2:** Key point detection methods.

**Model**	**Overview**	**Reference**
RSKDD-Net	Random sampling and random expansion clustering are adopted	Lu et al. (2020)
D3Feat	Combining salience and feature channel scoring to get key points	Bai et al. (2020)
PACNet	Learning weights through point nearest neighbor learning	Ko et al. (2018)
VK-Net	Generating key points in point clouds with different views of the same category	Chen et al. (2021)
DeepVCP	Predicting point saliency using a multilayer perceptron	Lu et al. (2019b)
PRNet	Two-parameter for features Number distance defines the significance of point	Wang et al. (2019)
IDAM	Predicting point saliency using a multilayer perceptron	Li et al. (2020a)
UCKD	Select the final key points according to the competency criteria	Ghorbani et al. (2022)

#### 3.1.3 Outlier rejection

Outlier pairs are inevitable in the actual registration process. Even in the most advanced registration methods, the removal of outlier pairs is a critical task. The existence of outliers will significantly reduce the performance of registration. The reasons for this are as follows: noise, outliers, partial overlap of point clouds and insufficient local features of point clouds. Removing outliers before solving rigid transformation for point cloud registration methods based on deep learning is often necessary.

In 3DResNet (Pais et al., 2020), researchers used a residual neural network to divide noise points into internal points and external points. In this way, the network acceleration can be realized without affecting the registration accuracy when outliers exist. However, 3D point cloud data has rich topological information and geometric features, so it is difficult to obtain more accurate registration results simply by dualizing it. Similar to 3DResNet, Yang et al. use compatibility features (CF) (Yang et al., 2021) to classify point pairs. The network first uses different points to check the compatibility of angles, lengths, etc., so as to obtain the compatibility scores of different points. Then, the information is aggregated to obtain compatibility characteristics. Finally, the feature number is input into the MLPs for intensive binary processing, which is still divided into inner points and outliers. Therefore, it does not break out of the shackles of the binary point of no use to enrich the information.

Spatial consistency due to Euclidean transformation between point clouds has received little separate attention in previous deep point cloud registration techniques. Therefore, Bai et al. (2021) proposed PointDSC to prune outliers by combining spatial consistency. First, a nonlocal feature aggregation module weighted by feature and spatial coherence is proposed for embedding corresponding features into the input. Secondly, the model is trained under paired space compatibility supervision. A differentiable spectral matching module is built to estimate the inner confidence of each correspondence relationship from embedded features. Finally, KNN is used to find the set of point pairs satisfying spatial consistency to solve the rigid transformation. Compared with the method of directly classifying point pairs, the outlier pair removal performance of PointDSC is greatly improved. In addition, the outlier pair removal module is embedded in many end-to-end models. The methods for removing outlier pairs are summarized as shown in Table 3.

**Table 3:** Outliers rejection methods.

**Model**	**Overview**	**Reference**
3DRegNet	Predicting point pair weights using binary classification networks	Pais et al. (2020)
CF	Dichotomous classification using MLP	Yang et al. (2021)
PointDSC	Combining spatial consistency to prune anomaly pairs	Bai et al. (2021)
DGR	Predicting point pair weights using binary classification networks	Choy et al. (2020)
IDAM	Calculating weights for predicting confidence in point pairs	Li et al. (2020a)
RPMNet	Using iterative normalization to obtain a double random matrix	Yew and Lee (2020)

#### 3.1.4 Motion estimation

In point cloud registration based on correspondence relationships, rigid motion attitude estimation is generally the last stage of point cloud registration. Motion parameters have different expressions, such as quaternion, angular axis, etc. The most common ones are undoubtedly rotation matrix and shift-vector. This method can be optimized by SVD based on correspondence relation, so it is widely used in point cloud registration methods of deep learning. At the same time, based on the end-to-end learning strategy, some motion estimation methods using regression strategy are also proposed. Since most of the methods differ in the way they use SVDS, this section summarizes and discusses existing methods only from the perspective of use.

PointDSC (Bai et al., 2021) divides network registration into training and testing stages. The weighted SVD is used in the training of the network, and the weighted estimated rigid transform based on least squares is used in the measurement. PRNet (Wang and Solomon, 2019b) uses confidence ranking for points, and selects point pairs with high confidence for SVD solution. Deep closest point (DCP) DCP (Wang and Solomon, 2019a) and DeepVCP (Lu et al., 2019b), the closest virtual point of depth, are calculated using the weighted confidence and the position of the point to solve the relative attitude. DGR (Choy et al., 2020) uses the weighted SVD to solve the rigid transform after passing the confidence selection point pair and retaining the confidence as the weight.

If only the point pair is entered into the solver module of the SVD, this usually means giving up the confidence of the point pair. This also causes the need to use coordinate values for network backpropagation. The coordinate values of point pairs are generally constant, which may hinder the propagation of gradient information in the network. While the reserved confidence allows the gradient propagation of the network's right to use weight value, and the right to use weight means the processing of the pair of points that do not exist in the target point cloud, which undoubtedly makes the network more dependent on the confidence estimation. The retained confidence of DGR model selection and the weighting method adopted by PointNetDSC certainly provide a new way to use SVDS. How to choose the confidence degree is worth thinking about in the future.

### 3.2 End-to-end

This type of network is referred to as an “end-to-end” network because it is trained to directly solve the registration problem by taking two point clouds or preprocessed point cloud data as input and generating motion parameters as output. The end-to-end network can preprocess the point cloud by integrating modules such as feature extraction, key point matching and outlier removal to obtain excellent correspondence relationships, and directly summarize all registration processes on the net-work. This section provides a detailed summary and discussion of the end-to-end net-work models that have been published as of this writing.

Lu was equal to the deep virtual counterpart DeepVCP (Lu et al., 2019b) proposed in 2019. In this network, a matching point generation mechanism is designed and feature descriptors of the mini-PointNet structure are used to extract the matching points to solve the problem of sparse point cloud. After the key points of the scene are extracted, the weights can be generated dynamically through the local features of the point neighborhood, and the positions of the key points can be fine-tuned. The weight of key points is combined in the network and the SVD operator newly introduced in TensorFlow is used to perform a single optimization iteration to construct another corresponding point. The distance between the newly generated corresponding point and the key point is taken as an additional loss function to conduct training supervision on the network. Moreover, global geometric constraints are added to the loss function to ensure its validity. This model can avoid the interference of dynamic objects and obtain high precision registration results. It is an effective deep learning improvement over ICP method. However, it still requires approximately accurate initial position between the target point cloud and the source point cloud, which belongs to the acceptable registration method. Cattaneo et al. (2022) proposed a new LCDNet. This model extracts features through the point voxel neural network (PV-RCNN) (Shi et al., 2020), which ensures that the model can process the point cloud data of large-scale scenes. At the same time, the Sink-horn method (Sinkhorn, 1964) is adopted in this model to realize unbalanced optimal transmission (UOT) in a differentiable way (Hori, 2002). UOT guarantees that the method can effectively match features extracted from the two point clouds, reject outliers, and handle occlusion points, while still being able to train the network end-to-end.

Based on the robust point matching method (RPM), Yew and Lee (2020) proposed an improved RPMNet method combined with deep learning. The model adopts the same feature extraction module as PPFNet, and adds 3D coordinates to obtain mixed features. By constructing Sinkhore structure combined with annealing method, virtual feature points are learned from mixed features containing spatial location and local structure information. The soft matching relationship is constructed based on this method, which significantly improves the registration accuracy under the condition of noise and low overlap. However, as an iteratively optimized network, the model needs to calculate the mixed features repeatedly in each iteration, which undoubtedly increases the computational cost of the method.

Considering that outliers may cause the network to fail to close the loop, the self-supervised network model with consistent cycle loss function may not be able to solve the registration problem of partially overlapping point clouds well. For this reason, Jiang et al. (2021) proposed CEMNet, an unsupervised sampling network guided cross-entropy point cloud registration method. The model consists of sampling network module and differentiable cross entropy (CEM) module. The registration task is transformed into a Markov decision process (MDP). The prior Gaussian distribution on space is learned through a sampling network, and initial data is provided for subsequent CEM modules. In the CEM module, each sampling transform is evaluated by combining the current and future new fusion reward score functions. At the end of the network, the future reward function is estimated by performing ICP methods on the transformed source and target point clouds. In this model, the differentiability of CEM is realized by top-k selection based on ranking. The loss function (Barron, 2019) based on scaling Geman-Mcclure estimation is used to train the network, and the sublinear convergence rate of outliers is used to reduce the negative impact of outliers on registration accuracy.

DWC (depth weighted consistent global registration Network) is also adopted in an unsupervised way (Ginzburg and Raviv, 2022). DWC first extracts the rotation invariant descriptor (RI) from the source point cloud through the factor extraction network, and then uses two variants of DGCNN, namely classification network DGCNNglob and segmentation network DGCNNloc, respectively to achieve feature extraction. Then cosine similarity is used to define a soft sampling map, sample K pairs of points with corresponding relationships and calculate the rigid transformation, and then select the rigid transformation of the internal points with the highest confidence. Finally, the weighted consistency loss function is used to train the optimal network. Compared with the supervised approach, the unsupervised approach avoids the large amount of annotated data required for the training process and the associated computational costs required for the training, making the model significantly faster.

Inspired by traditional point cloud registration methods, many methods choose to construct deep convolutional networks as key point detectors or local feature descriptors of point clouds. For example, DCP constructs key point matching in the scene by learning the features of the point cloud neighborhood and combining the attention mechanism. PR-Net (Wang and Solomon, 2019b), on the basis of DCP, adopts the actor-critic model and uses the feature of global aggregation point by point to obtain the global feature. Furthermore, Gumbel-softmax (Jang et al., 2016) method was combined to improve the registration accuracy of the model in the low-overlap scenario. Unlike the above two methods of constructing key points in the point cloud, DeepGMR (Yuan et al., 2020), a deep Gaussian mixture model registration method, adopted a registration method based on global features. In this model, the postural invariant correspondence between source point cloud and Gaussian mixture model (GMM) parameters is extracted by neural network, and the registration formula is modeled into the minimum divergence of Gaussian mixture probability distribution by maximum likelihood framework. GMM parameters are calculated using differentiable computing module and the optimal transformation is recovered. The model has strong resistance to the initial pose and noise of the point cloud, and does not require iterative optimization. To overcome the insufficient information caused by the direct aggregation feature of deep learning point cloud registration based on PointNet, Kurobe et al. (2020) proposed the Communication network (CorsNet). By connecting the global feature with each point feature, the model feeds back to the local feature of each point to return the point cloud correspondence.

Different from the way of voxelizing point cloud, Ali et al. (2021) proposed RPSRNet, which used the tree representation based on Barnes Hut (BH) (Barnes and Hut, 1986) for the input point cloud data. First, the model recursively subdivides the normalized boundary space of the input point cloud to the limit depth to construct the BH tree. With the help of the established index of the tree and the hash graph (Bader, 2012), the network can retrieve the neighborhood portion of a given node. Secondly, the layered feature extraction (HFE) module is embedded in the model for global feature extraction. Finally, differentiable SVD is used to solve the rigid transformation. The model has achieved good results in processing speed and accuracy. However, there are limitations in the registration of partially overlapping point clouds.

To register real data, Choy et al. (2019) proposed an end-to-end network for deep global registration (DGR). Firstly, the FCGF (Choy et al., 2019) structure is used in the feature extraction stage. Still, the 6D convolutional network of the Minkowski engine is used to predict the confidence when the pair of outliers is removed. At the same time, the micro-weighted Procrustes method (Gower, 1975) and the robust gradient-based optimizer (Zhou et al., 2019) are used in this model. As an end-to-end network, the model allows users to replace the registration parts with existing plugin modules. This is one of the great benefits of an end-to-end network.

In the registration method based on point matching relation, the most important is to obtain reliable correspondence. Usually, the results obtained by feature extraction and matching are only sometimes reliable. Therefore, detecting key points and removing additional outlier pairs is necessary. This makes end-to-end networks that integrate various functional modules increasingly popularly. The integration of multiple modules brings not only the improvement of registration performance, but also the scale and complexity of the network. As a result, the model has higher and higher requirements on hardware, and it is tough to process point cloud data in large-scale scenarios.

### 3.3 Registration method based on global feature

**Figure 4:**
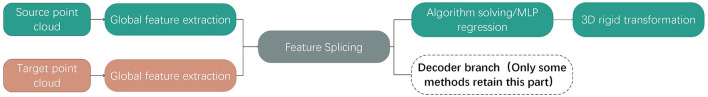
Flow chart of the global feature-based registration method.

Currently, the mainstream complete overlapping point cloud registration is generally based on the corresponding point matching relationship. But in addition, the researchers also explored another uncorrelated point cloud registration method based on global features. The main component of this method is to search the difference of global features between two input point clouds and process the extracted point cloud features by a pooling layer. The influence of the sequence of point pairs on subsequent registration is removed while preserving the global features. Finally, the global features of source point cloud and target point cloud are spliced, and then the parameters required for rigid transformation are solved by MLP regression or other methods. The registration solution method based on global features is shown in Figure 4.

Aoki et al. (2019) first proposed a global registration method PointNetLK based on PointNet coding rules. In this method, global features of point cloud are extracted by PointNet and Jacobian matrix of global features of point cloud is calculated by inverse synthesis formula. The Lucas & Kanade (LK) method is used to solve the rigid transformation from global features. Sarode et al. (2019) proposed an unsupervised method PCR-Net based on PointNetLK using MLP regression rigid transformation to solve the problem that PointNetLK is sensitive to noise. The model represents the three-dimensional rotation as a quaternion and uses Siamese-like network architecture to predict motion parameters. Meanwhile, Chamfer distance loss function is used to constrain network training. Higher accuracy is achieved in the complete overlapping point cloud registration. At the same time, it also has good robustness in noisy scenes. AlignNet3D network (Groß et al., 2019) through the design of the CanonicalNet module. The input point cloud is transformed into another attitude in advance, then the global characteristics of the point cloud are learned and output through MLP structure, and the network structure similar to PCRNet is utilized to learn and output attitude transformation.

Deng et al., inspired by PPFNet, have put forward a data-driven point cloud registration network PPF-FoldNet by incorporating the self-coding module of Folding structure into the network. The network can learn to arrange features independent of rotation unsupervised. On this basis, a RelativeNet module is designed to predict relative attitude directly for registration. This method shows a high recall rate in real data sets. However, because there is almost no accurate matching relationship in the actual point cloud, this method shows low registration accuracy due to the problems such as noise and low overlap. In order to deal with the influence of non-overlapping points, Xu et al. (2021a) proposed OMNet. In each network iteration, the overlap mask between source point cloud and target point cloud is predicted respectively to mask the non-overlap area, the partial overlap problem is transformed into a complete overlap problem, and the motion parameters between two point clouds are predicted by MLP module, which has achieved the most advanced effect at present.

The feature measure registration method (FMR) proposed by Huang et al. (2020) uses the cod-decoding model to supervise the global feature registration. The coordinate information of the input point cloud is restored through the decoder region to achieve the effect of eliminating redundancy of extracted global features and ensuring that the information is not lost. RANSAC is not required for global feature registration by this method, and the registration speed is improved to some extent compared with the method of removing outlier pairs. At the same time, this method achieves excellent results in real data sets and cross-source point cloud registration. Unlike the form of establishing key point matching relationship, the deep learning point cloud registration network based on global features starts from the overall structure and directly perceives and codes the attitude information of the point cloud without establishing key point matching. Therefore, this kind of method runs faster. However, such methods have received less attention, mainly because such methods perceive features based on the overall form of point clouds. When the degree of overlap between input point clouds is low, the information of non-overlapping areas will significantly interfere with the perceived pose information. Therefore, the registration method based on global features is more sensitive to the overlap degree of point clouds.

## 4 Incomplete point cloud registration

The performance of complete overlapping point cloud registration is approaching saturation levels under current evaluation criteria and existing datasets with the introduction of increasingly sophisticated techniques. However, the sparsity, low overlap rate and random distribution of real data make it difficult to establish accurate and stable correspondence relationships with fully overlapping deep point cloud registration methods. In particular, when point cloud registration is actually applied to various fields, it is inevitable to encounter low overlap degree of point cloud to be registered. This creates new challenges for existing networks and methods. This article summarizes and reviews the existing deep learning partial overlapping point cloud registration methods in detail. According to the processing method of non-overlapping point cloud, the registration method of partial overlapping point cloud based on deep learning is divided into the method for non-overlapping region, without considering the method of non-overlapping region.

### 4.1 Regard overlapping regions

Point cloud data frequently can only describe the part of the object/scene that is visible to the sensor and not covered by occlusion. In addition, sensor noise, reflective surfaces, or artifacts can sometimes create points in the point cloud that do not correspond to any surface in the object or scene. Missing data and irrelevant points in point cloud pose new challenges to point cloud registration methods. The method of removing outlier pairs mentioned above cannot cope well with the registration of point clouds with low overlap rate. Moreover, the calculation cost of the specialized outlier removal module is high, so it cannot be applied to real scenes in real-time. To this end, researchers have proposed a series of new methods for partially overlapping point clouds.

#### 4.1.1 Reject non-overlapping regions

Sarode et al. (2020) proposed a full convolutional neural network MaskNet. Perform learn-based inner estimation based on the PointNet structure. The network is trained to identify which points in the target point cloud are internal by calculating masks, so that these points and the source point cloud closely describe the same part of the object/scene geometry. Then the network can “shield” the outlier area from the target point cloud. This method can deal with partial overlapping point clouds and outliers well, and also has a specific effect on noise suppression. But MaskNet is currently limited to removing points from a cloud of input points, which limits its practical application to scene reconstruction. Also, because the network uses PointNet coding, a large amount of labeled data is required for training. Shotton et al. (2013) proposed Overlapping Mask Network-OMNet, an iterative end-to-end network based on global features, aiming at the overlapping area point cloud. In each iteration of the network, the overlapping mask of two input point clouds is predicted respectively. Given an exact overlap mask, non-overlap points are rejected during the aggregation of global features. This converts the part-to-part assignment to the same shape registration. This method removes the non-overlapping regions to obtain the global features without interference, which makes the regression rigid transformation easier. It reduces the sensitivity of the registration method to the initial position of the input point cloud. The robustness of the method to noise and pose deflection is also enhanced. In addition, the data used for the previously proposed network model is sampled only once from the CAD model of each object, resulting in the same source and reference point clouds. OMNet proposes a more practical approach to data generation in which the CAD model is sampled twice as a source and reference, avoiding the overfitting problem that was prevalent previously. However, this model does not consider the impact of the amount of point cloud data, and can only be applied to the scene or object with a small amount of data.

Since most network models usually use the inner product of features or the norm distance of features to express the matching degree between two points, this method undoubtedly ignores the differences of features in each channel. To this end, Li et al. (2020a) proposed the iterative range-aware similarity matrix convolutional network (IDAM). The model combines geometric features and distance features into the iterative matching process to solve the problem of fuzziness. A two-stage point elimination technique is used simultaneously, filtering out points that are unlikely to match the confidence level in the first step, and eliminating point pairs rather than single points in the second step by mixing points. The learning network is trained with weak supervision by assigning weights to false positive point pairs. This network can be compatible with traditional point cloud feature extraction methods such as fast point feature histogram FPFH (Rusu et al., 2009) and graph convolutional neural network GNN (Shi and Rajkumar, 2020). IDAM has significant computing advantages in the case of dense point clouds. However, the network relies heavily on selecting key points to avoid outlier pairs, so the registration result depends on the detection of the network key points.

Based on DCP, Wang and Solomon (2019b) proposed a partial-allocation quasi-network (PRNet). One of the key new components is the partial key point detection module. The network model converts partial overlap into detecting the common points of two point clouds, removes non-overlapping areas, matches these key points, and solves the Procrustes problem. The model samples keypoint correspondence using Gumbel-SoftMax and a pass-through gradient estimator. The framework allows coarse matching using diffuse (fuzzy) matching for far-point clouds, and the final refinement iteration becomes clearer. At the same time, the model does not introduce additional hyperparameters, but uses an embedded subnetwork to predict the annealing parameters and convert them into a simplified version of the actor-evaluation model. Finally, iteration optimization technique is used to improve the registration accuracy. The model achieves good results in low overlap and noise scenarios, but its practical application is limited due to the large scale of the network. At the same time, the input point cloud should have a unique local geometric structure to extract reliable sparse 3D feature points.

Using the network to learn highly unique point features of different locations but similar shapes is challenging. In order to solve this problem, Min et al. (2021) put forward Geometry Guided Network. The model uses a spherical position coding method and self-attention mechanism to assign global geometric position information to irregular 3D points to learn global unique features. And assign this feature to each point. The uniqueness of each feature is further enhanced by the unsupervised geometric consistency loss function. The model uses two normal forms to predict the rigid transformation: unsupervised geometric consistency loss, which is used to minimize the geometric distance between the transformation source and the target point cloud; By having a supervised transformation loss, which is used to estimate the rigid motion itself. The proposed method is robust in partially overlapping and noisy registration. However, since the model assigns globally unique features extracted to each point, it requires the enhancement of geometric uniform loss function. However, the parameters of the subnetwork loss function are not predicted, so the optimality of the parameters cannot be guaranteed. At the same time, the selection of spherical position coding coordinate system also greatly influences the performance.

In practice, point clouds vary in size due to capture distance, sensor type, environment, and many other factors, making it difficult for traditional methods to cope with this situation. To this end, Yan et al. (2022) proposed a partial feature extraction network PointpartNet. It is a neural network that divides the point cloud by KNN, extracts the features of each part, and calculates the matching likelihood score of each part. The matching likelihood score predicts which part of the larger intact point cloud is most similar to the smaller point cloud. This result is used to divide registration into global registration and local registration using SVDS to avoid falling into local minima. The model successfully registered point clouds of different sizes, which was almost impossible to achieve before. However, since the feature extraction module of the model uses a PointNet structure, it is affected by the initial direction. At the same time, the model has not been tested on a real scene, so there is still a distance for its practical application.

In order to register poorly initialized and partially overlapping point clouds, Li et al. (2021) proposed a Deep Weight Global Registration network, DWG-Reg. Firstly, the bidirectional nearest correspondence strategy is introduced to establish a reliable symmetric registration model. Secondly, aiming at the problem of partial overlap, a weighted pruning strategy is proposed to filter the wrong correspondence of points, and a probabilistic method is proposed to suppress the noise. A secondary network is introduced to predict the optimal annealing parameters, and a Hybrid distance generator network is used to learn the mixed distances of corresponding points, and a robust kernel function is used to estimate the corresponding confidence. Finally, a weighted optimizer is introduced into the network to solve the registration problem.

#### 4.1.2 Prediction complements non-overlapping regions

In addition to the above network model of eliminating non-overlapping regions, the researchers have proposed a series of methods to predict and supplement non-overlapping regions. Based on D3Feat and combined with Transformer attention mechanism, Huang et al. (2021a) proposed a point feature extraction method PREDATOR. The self-attention and cross-attention mechanisms are used interchangeably in this method, so the local and global information of point cloud can be obtained simultaneously. At the same time, thanks to the predicted overlap region probability and the unique feature matching point selection logic, the method can achieve good results in the scene with 10 to 30% overlap. However, the registration accuracy of this method depends heavily on the accuracy of matching points, so the local features of the input point cloud must be significant. Otherwise, the extracted features are more generalized, easily leading to mismatching.

To solve the above problems, Zhang et al. (2022) proposed a two-stage partially overlapping point cloud registration method based on global features. Firstly, the Edge-Conv layer is used to map the input two point clouds to the high-dimensional space. Since the characteristic information interaction between two point clouds is necessary, the model uses a micro-overlapping region prediction module with an attention mechanism to predict the overlapping region. Finally, the sampled point cloud is taken as input, and the attention mechanism is used to capture the global information of the point cloud. The so-called two-stage registration of this method means that in the first stage, edge convolution is combined with Transformer to predict overlapping areas to improve the global feature quality in the next stage. In the second phase, PointNet is combined with the attention mechanism to capture global information and use it for robust regression. The model deals with point clouds from different sources and target point clouds with uneven density and obtains excellent results. However, in the face of over-fitting of training data, the registration accuracy of the model will be reduced. At the same time, this model is not an end-to-end network, and cannot deal with large-scale point cloud data.

In addition, Žagar et al. (2022) proposed a point cloud registration method centered on target objects in view of the impact of non-overlapping regions in feature extraction by global feature-based registration methods. First, the most distant point sampling (FPS) is used to stratify the input point cloud to extract the object of interest. Due to the self-occlusion of the 3D sensor, the extracted points can only partially represent the object of interest. Then PointTr (Yu et al., 2021) was used to predict the missing points and increase the similarity between the extracted point clouds. Using this similarity, the completed point cloud of the object of interest is roughly aligned. Finally, the registration on the entire capture scenario is refined by using roughly estimated transformation parameters as initial conditions. By focusing on object centric alignment, the model can overcome the problems of point clouds captured from different viewpoints, low overlapping point clouds, and the need to learn correspondence across the entire scene.

Consistent Two-Flow Network (CTF-Net) is proposed by Yan et al. (2021). Considering that the completed shapes are easier to register than each shape individually, and the degree of overlap of the complete shapes increases, the registration becomes easier. Therefore, the method uses neural network to learn the prior knowledge of a class of objects and completes the shape completion. The main idea of this model is to train two independent networks jointly to complete the two tasks of non-overlapping part shape complement and point cloud registration after compliment. The model uses two coupled flows to train the registration network and the completion network simultaneously. One network performs registration-completion, and the other performs completion registration to maximize the consistency of both registration and completion. Since the completion network in the method only generates the information of the missing part, the completion along the two branches of the network may be different. A particular conformance term is set up for this method to generate the completion of the specification. This method performs well when point cloud overlap is low or even non-existent. Thanks to the prior knowledge learning of neural networks, the original complete shape can be restored from the local shape. However, the completion strongly relies on the prior knowledge brought by the training set, which brings challenges to the type and quantity of data, and also brings great limitations to the popularization and generalization of the method. At the same time, due to the fuzziness of the completion task itself, the registration without overlapping is also complicated.

To deal with the influence of non-overlapping regions on registration results, researchers conducted a series of studies from the positive and negative directions of removing and predicting the addition of non-overlapping regions. The method of eliminating the non-overlapping area can theoretically be applied to the various methods of complete overlap in order to deal with partial overlap. But the degree of overlap is low, unless the overlap area greatly affects the final registration result, or even mismatch. However, due to its late start, the current method does not have an end-to-end net-work, so it cannot deal with large-scale point cloud data, which limits the further practical application of this method. Registration methods for non-overlapping regions are summarized as shown in Table 4.

**Table 4:** Summary of registration methods for non-overlapping regions.

**Category**	**Model**	**Reference**
Reject non-overlapping areas	MaskNet	Sarode et al. (2020)
		OMNet	Xu et al. (2021a)
		IDAM	Li et al. (2020a)
		PRNet	Wang et al. (2019)
		Geometry guided network	Min et al. (2021)
		PointpartNet	Yan et al. (2022)
		DWG-Reg	Li et al. (2021)
Prediction non-overlapping areas	PREDATOR	Huang et al. (2021a)
		Two-stage partial point cloud registration	Zhang et al. (2022)
		Object-Centric Alignment	Žagar et al. (2022)
		CTF-Net	Yan et al. (2021)

### 4.2 Disregard non-overlapping regions

Registration becomes extremely difficult when the overlap between two point clouds is extremely small. For the registration network, if its influence on the overlapping region and noise are extremely robust, there is no need to carry out additional operations on the non-overlapping region of the point cloud. Therefore, researchers propose a series of deep learning point cloud registration methods that do not require processing of non-overlapping point clouds.

The strengthening of the network itself mostly starts from the direction of information acquisition or noise suppression. Therefore, this article only summarizes and discusses each method from the perspective of implementation. Huang et al. (2020) proposed a global registration method of three-dimensional point cloud based on decoder, FMR. The coordinate information input point cloud is restored by decoder to achieve the effect of eliminating redundancy and information loss of global features extracted from input point cloud. Registration by global features does not require RANSAC, so it has a greater advantage in speed than removing mismatched points. Lu et al. (2019a) proposed a simple network based on spatial and channel attention called SCANet. The spatial self-attention aggregation (SSA) module is used in the feature extraction subnetwork to effectively utilize the internal information and global information of different levels of input point cloud. The channel cross attention regression (CCR) module is used in the sub-network of pose estimation to realize the information interaction between two input global feature vectors, enhance the correlation information and suppress the redundant information.

Because the point cloud data is easily polluted by noise in practical application, the point cloud density of the overlapping part is also different. So it's hard to find a dot correspondence. In order to overcome these problems, Xie et al. (2022) proposed a new approach based on Siamese architecture, namely, end-to-end micro-depth network convolution with Siamese point network (CSPN). A pyramid structure is proposed to learn multi-scale features of each point. This structure can extract local features and combine features of different levels for point cloud registration. At the same time, the model uses a new matching matrix calculation method based on the robust point matching method RPM, using information extracted from the feature space and coordinate space. Combined with the unique attention mechanism to deal with the conflict between coordinate matching matrix and feature matching matrix, the influence of mismatching points is reduced.

Recent hybrid feature approaches improve high point cloud registration performance by emphasizing more integrated information (Li et al., 2020a). However, mixed feature extraction often ignores the dimensional difference, semantic gap and mutual interference between shape features and spatial coordinates. Therefore, Wang et al. (2022) proposed a new multi-feature guidance network (MFGNet) to overcome the inherent defects of mixed features. The network combines spatial coordinates and local features to guide the corresponding search. In contrast to the previous approach using mixed features, MFGNet consists of two distinct branches of matching matrix computation: coordinate matching matrix computation and feature matching matrix computation. The two branch learning networks independently assign the correct correspondence. The final matching matrix is obtained by blending the two matching matrices, and the differentiable singular value decomposition (SVD) layer is used to solve the rigid transformation between point clouds. In addition, in order to deal with the conflict relationship between two matching matrices, the corresponding credibility computing module scores the reliability of each pair of responses, thus significantly reducing the impact of mismatches or mismatching points. The experimental results on the data set show that the method can still achieve excellent accuracy and robustness under the condition of invisible point cloud, unknown class and noise. However, the method does not use the attention mechanism to calibrate the matching results, nor does it use the new loss function to supervise the learning of the network, so there is still a lot of room for improvement. In order to deal with the above problems, SLORNet (Li et al., 2020a) integrated the attention mechanism into the network model, weighted the pose information perceived by different positions, formed a channel for information interaction between scenes, and achieved good results in scenes with low overlap.

The deep learning point cloud registration method using singular value decomposition (SVD) to find the rotation matrix does not fully consider the scale information, and it is difficult to deal with the point cloud with large initial rotation Angle. For this purpose, Zhang et al. (2021) proposed a High-Dimensional Regression Network (HDRNet). Firstly, the 3D point cloud is mapped to a higher dimension through the feature extraction layer. Then the corresponding matrix estimation layer is used to learn to express local features explicitly. Secondly, the corresponding matrix is embedded in the feature embedding and fusion layer. It includes calculating the inner product matrix of local features of two-point cloud. This method fully improves the characteristics of source point cloud and can effectively handle the registration of point cloud with large initial Angle. The method then uses 2DCNN to further extract the features and extend them to one-dimensional tensors. Finally, a scaling factor is introduced at the end of the fully connected layer and the tensor is used to return the attitude information. In addition, the EMD loss function in PCRNet is used to train the network, which ensures that the method can cope with multi-scale partial overlapping point cloud registration. However, since the method only uses the improved loss function to deal with partially overlapping point cloud registration, the registration accuracy of point cloud will decline rapidly when the local features are not obvious.

The degree of overlap between the target point cloud and the target point cloud is an important factor affecting the practical application effect of point cloud registration. Although the recently proposed partial point cloud registration network of deep learning has designed a unique structure combined with its own method characteristics to alleviate the interference of non-overlapping regions, it is far from being able to completely eliminate the influence of non-overlapping regions. A summary of the methods in this class is shown in Table 5. In the future, more innovative networks are needed to solve the problem of point cloud registration for large-scale, low overlap and real-time applications.

**Table 5:** Method of disregarding non-overlapping regions.

**Category**	**Model**	**Reference**
Disregard non-overlapping regions	FMR	Huang et al. (2021a)
		SCANet	Lu et al. (2019b)
		CSPN	Xie et al. (2022)
		MFGNet	Wang et al. (2022)
		HDRNet	Gao et al. (2022)

## 5 Network acceleration

Deep learning-based point cloud registration methods have made great progress, but the existing methods are mostly in the laboratory environment, using the existing data set for training, testing and horizontal comparison. How to apply the existing network model to the actual field and develop a real-time, accurate, and fast registration method is a problem that researchers have to think about. With the development of computer hardware technology and the emergence of advanced networks, the practical application of deep point cloud registration technology is becoming more and more. Therefore, this section will make a detailed summary and comment on the neural network acceleration application of deep learning from the hardware and software parts.

### 5.1 Hardware

The continuous development and breakthrough of GPU technology (Nickolls et al., 2008; Lee et al., 2010; Rosenberg et al., 2020; Gao et al., 2022) has ushered in the vigorous development of deep learning technology. Bakhoda et al. (2009) proposed a brand new high-performance GPU parallel space hash framework ASH. With this new framework, richer functionality can be achieved with fewer lines of code. Such as space transformation operation, geometric reconstruction and micro-appearance reconstruction. At the same time, the framework can index the data of direct access to outer space changes, and realize seamless integration with Pytorch and other neural network frameworks. For large-scale 3D sensing tasks such as point cloud voxel, scene reconstruction, non rigid point cloud registration (Jin et al., 2021; Dong et al., 2022), and volume deformation, researchers are allowed to complete the previous work with less code workload.

In order to achieve high accuracy in deep learning network, the scale of deep learning point cloud registration model is increasing. However, due to the limitation of single GPU memory, it is difficult to achieve high precision and large scale depth model. For this reason, NVIDA introduced unified memory technology in the sixth generation of CUDA to expand GPU memory through CPU memory and device memory (Harris, 2017; Chien et al., 2019; Choi and Lee, 2021; Lin, 2022). The data required for point cloud registration of deep learning can be roughly divided into three categories: parameters required by the model, input data and intermediate results. In each iteration run, new values are needed to update the parameters required by the model, and this work must be carried out on the GPU until the training is completed. The data access mode during registration is similar to the memory suggestion mode supported by NVDIA. Therefore, the unified memory technology of CUDA can more effectively implement the point cloud registration of deep learning. In order to cope with the limitation of single GPU, many institutions have built large GPU clusters for deep neural network (DNN) training (Sakharnykh, 2018). GPU clusters generally provide services for parallel tenants. Multi-user GPU clusters are usually managed by the cluster manager (Hindman et al., 2011; Vavilapalli et al., 2013; Wu et al., 2021) or the GPU cluster customized scheduler (Peng et al., 2018; Xiao et al., 2018; Gu et al., 2019; Mahajan et al., 2020). Users submit deep neural network (DNN) training tasks and their resource requirements, such as the number of Gpus and rental time, to the cluster. To realize the use of idle resources to complete the performance analysis, work migration and other goals. It also provides a new and efficient hardware foundation for deep learning point cloud registration researchers.

In addition to GPU, the development of new hardware, especially surface scanning, line scanning, active/passive laser, and femtosecond lidar, also provides new opportunities for point cloud data registration of Lidar (Zhang et al., 2017).

### 5.2 Software

In addition to the hardware mentioned above, software is also an important factor affecting the registration effect of deep point cloud. Most of the existing models strengthen the learning ability of the network through the complex design of the network structure, but ignore the complexity of the network operation and the time cost. Some methods can only be applied to small-scale point cloud data and cannot deal with actual large-scale point cloud data.

Therefore, recently researchers have proposed a variety of solutions for deep learning neural networks affected by point cloud data in large-scale scenarios. For example, RandLA-Net (Cheng et al., 2018) uses random sampling method to replace the nearest point sampling of iteration, random ellipsoid sampling and other methods that cost much calculation. Meanwhile, a pooling layer based on attention mechanism is designed to alleviate the influence of random sampling on learning mechanism. CSPN (Xie et al., 2022) adopted a pyramidal network structure to help extract and combine features of different levels. PVCNN (Hu et al., 2020) and improved PVRCNN (Shi et al., 2020) network combined the network structure of PointNet with the learning mode of 3D voxel grid structure, and designed PV-Conv as the basic module of the learning network. The PV-Conv structure can aggregate neighborhood features at low resolution through 3D-CNN of 3D voxel lattice structure. The PointNet is used to supplement the lack of information in high resolution single point mode. Thus, rapid perception and collection of point cloud features can be realized in large-scale scenarios. The newly proposed SPVNAS structure (Liu et al., 2019b) further improves PC-Conv. By combining with sparse convolution structure, it can accommodate voxel lattice with higher resolution and improve the computing efficiency of the network. At the same time, because some existing models are not end-to-end, combining traditional registration methods is necessary. This undoubtedly brings additional computing costs. Therefore, the development of end-to-end networks, or more easily integrated network modules, is also a valuable direction.

While ensuring the effectiveness of the method, real-time processing of massive point cloud data is an urgent problem for deep learning point cloud registration methods. If you want the solution to be real-time, as well as data processing in large-scale scenarios, you have to consider the support of computer hardware and software. In future work, more excellent performance of GPU and its running conditions, and more efficient method structure are undoubtedly the focus of attention of researchers.

## 6 Applications

**Figure 5:**
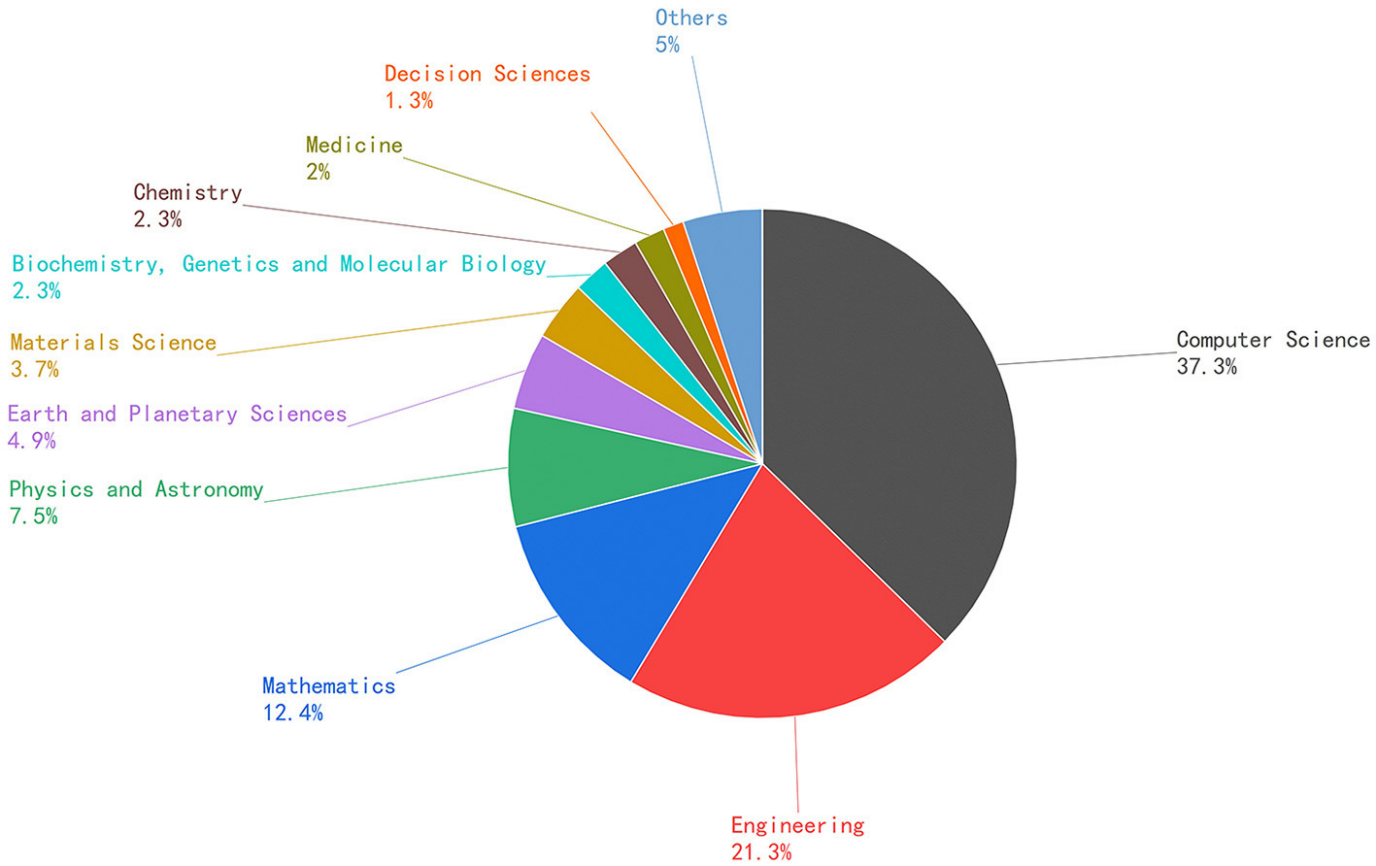
Interdisciplinary integration of deep learning-based point cloud registration (Source: Scopus database).

Point cloud registration plays a pivotal role in various practical applications, as illustrated in Figure 5, which depicts the integration of deep learning-based point cloud registration with disciplines such as computer science, engineering, mathematics, medicine, and more. This section will delve into the real-world utilization of deep learning for point cloud registration and offer insights into its application trends and prospects in these diverse fields.

### 6.1 Medical

Real-time medical image registration has always been a frontier problem in the medical field. This is especially true of prostate cancer, the second most malignant disease in the world. MR-TRUS image registration enables targeted biopsy and brachytherapy, resulting in accurate perineal biopsy needle insertion and brachytherapy catheter placement. Because the manual registration process depends on doctors, it is time-consuming and cannot be replicated. Therefore, Tang et al. (2020) proposed a nonrigid MR-TRUS image registration framework ProRegNet for prostate intervention. The registration framework consists of a convolutional neural network (CNN) for MR Prostate segmentation, a CNN for TRUS prostate segmentation, and a point cloud-based network for fast 3D point cloud matching. In addition, based on the simulated Fe-TRUS training, ProRegNet can predict the point cloud motion of the prefrontal glands under the implicit biomechanical constraint and achieve automatic MR-TRUS image registration.

In prostate cancer treatment, radiation reagents can be injected into major prostate lesions to control tumors effectively. However, it is difficult to identify major intracortical lesions on CT images. Multi-parameter MRI has superior soft tissue contrast and is commonly used to detect lesions in the oral cavity. For this reason, Fu et al. (2021a) proposed a deep learning-based point cloud matching network, which registered multi-parameter MRI and computed tomography CBCT images to identify the main lesions of radiotherapy for prostate cancer. CBCT and MRI first contour the prostate, which is then meshed into point clouds. This method trains the network using the point cloud generated by finite element analysis. The trained network can perform MRI-CBCT prostate image registration under biomechanical constraints, bringing new opportunities for identifying diseased sites.

Physical registration of nonrigid images is a critical component of image-guided liver surgery. To overcome the problems caused by noise, partial overlap, and sparse point clouds during the operation, Fu et al. (2021b) propose a new grid point cloud registration based on occupancy learning-based, which trains the point cloud depth network to reconstruct occupancy functions from sparse points. The reconstructed liver was used to guide nonrigid registration and to align preoperative liver images with intraoperative samples. Considering the booming development of the medical field and a large amount of research funds, it is expected that the field will remain strong in the practical application direction of deep learning point cloud registration (Jia and Kyan, 2021).

### 6.2 Autonomous driving and intelligent vehicles

Recently, point cloud registration is widely used in intelligent vehicle research, including creating larger 3D scanning scenes, map matching, visual odometer, attitude estimation, and other directions. Intelligent vehicles must recognize the exact location and category of surrounding objects in various situations to take into account interactions with them. As a result, light detection and range sensors called LiDAR (laser radar) are widely used in smart cars. LiDAR provides information in the form of point clouds that can be used to locate and classify surrounding objects. However, unlike vision-based object detection and classification systems, LiDAR point clouds do not have enough shape information to classify dynamic objects due to the sparsity of points. To solve this problem, Min (2019) proposed a brand new model, which enhanced classification performance based on deep learning by adding LiDAR point cloud shape information. A hierarchical accumulation method considering three degrees of freedom motion of dynamic objects is also used. The test on actual vehicle data obtained excellent results. However, the classification performance of this model will decline with the increase of nonrigid objects such as pedestrians.

Loop closure detection, as an essential component of the SLAM system, can reduce the drift accumulated over time. Cattaneo et al. (2022) proposed a new registration method for loop closure detection, LCDNet, which can simultaneously perform loop detection and point cloud registration. LCDNet estimates the whole six degrees of freedom (DoF) relative transformation between point clouds under driving conditions, which is completely different from previous registration methods and helps to achieve faster convergence in subsequent ICP refinements. This method can also be integrated into the existing Lidar SLAM database (Kim et al., 2021).

In addition, point cloud registration between real-time point cloud of vehicles and 3D maps can be applied to real-time vehicle positioning. The critical requirements of autonomous driving for the positioning function are high precision and real-time efficiency. The development of a high-precision and fast registration method with prior road information is the research direction of automatic driving (Shan et al., 2020). Meanwhile, the existing image-to-point cloud registration methods mainly aim at vehicle platforms along paved roads. The image-to-point cloud registration on the UGV platform suitable for off-road driving is also a new application direction (Li and Yang, 2021).

### 6.3 Architecture

BIM (Building Information Modeling), as a new generation of information storage and operating system, is widely used in architecture and construction management. The system usually contains a three-dimensional model and properties of the building. Previous computer-aided BIM designs were only capable of simple guidance and theoretical planning because there was no linkage between the system and the real world. Point cloud data can overcome this limitation and precisely align the digital model with the physical space. Significant advances in 3D imaging technology enable us to effectively monitor and manage engineering progress during construction (Omar and Nehdi, 2016; Jeon and Seo, 2022). The application of point cloud data will make it easier to evaluate, visualize and transform projects dynamically. In general, point cloud data can be used with BIM to visualize the progress of the project. Yang et al. (2015) proposed an automatic registration method based on the deep learning method of a data-driven convolutional neural network (CNN). The model can identify the differences between the “planned” and “actual” models with greater accuracy, ensuring that monitoring and construction can be carried out in real time. Sheik et al. (2022) proposed a method of using corner points to register the scanning model of buildings with the BIM model. Using Building SMART International Ltd. based on IFC data exchange format, they directly employed the IFC schema to extract lossless geometric details, rather than converting BIM to another format. In addition, indoor and outdoor point cloud registration is also a challenging research direction (Thein, 2011).

Although point cloud data brings a new level of technological innovation to the construction industry, two obstacles limit its widespread use. First, 3D sensors are expensive. Secondly, the existing registration methods could be more efficient, and the main factor lies in the slow speed of registration methods. Most of the current methods are improvements on the traditional point cloud registration (Kim and Cho, 2017; Kim et al., 2018; Lei et al., 2019). However, there is still a lack of more robust and fast deep learning point cloud registration methods in the field of building information. In future research, it is urgent to develop a fast and high-precision registration method that can be applied in architecture. The combination with deep learning point cloud registration is also a new development opportunity in architecture.

### 6.4 Industrial

In the field of automation-related industry, in order to overcome the shortcomings of manual assembly, automatic assembly system has developed rapidly. Zhang et al. (2012) proposed a flexible and unified system. The system integrates a partial point cloud registration architecture, including deep learning-based and iterative nearest point (ICP) methods for rough and exact registration, respectively, to solve time-consuming matching problems. At the same time, it also includes an open loop, closed loop, visual-based control, and force control required by the semi-automatic assembly, so as to solve the problem of inconvenient manual operation in industrial manufacturing.

The blade is known as the crown jewel of modern industry, widely used in aeroengine, steam turbines, wind turbine, etc. In order to ensure perfect and stable aerodynamic performance, the modern industry requires very high dimensional accuracy and surface integrity of the blade. An accurate measurement of blade profile is a key means to guide blade production. At present, non-contact optical measurement technology is particularly prominent in blade profile measurement. A fundamental problem of this technology is how to arrange measurement point clouds from different viewpoints into a complete profile. Xie et al. (2022) proposed a new general model CSPN for blade profile registration on the basis of the developed multi-axis motion system. With the learning ability of the deep neural network, the accuracy of registration is greatly improved.

In addition to the above applications, Peng et al. (2023) presents a novel approach to modeling soft fabric-type actuators using deep learning to correct simulated point clouds and improve their resemblance to real actuators. By employing PointNet and LSTM, the authors effectively capture time-series data and enhance the accuracy of point cloud features prediction, ultimately improving the similarity between corrected simulated point clouds and real data. The integration of deep learning into this context is promising for applications in robot design and control, enabling rapid and cost-effective real-time simulations of wearable devices.

### 6.5 Other applications

In addition to the above fields, researchers have been exploring the application of deep learning point cloud registration technology in other fields in recent years (Peng et al., 2022; Mao et al., 2023).

In many countries, the protection and restoration of cultural relics have attracted more and more attention [CHANGE project & PRESIOUS project]. As one of China's most precious cultural relics, excavating and preserving the Terracotta Warriors has always been a major challenge. Many of the terracotta warriors exist in fragments, and reassembling them manually would be laborious and time-consuming. For this reason, Chang et al. (2021) proposed a recombination method SPPD based on the fracture surface. It provides a precious tool for virtual restoration of three-dimensional cultural heritage. Augmented reality (AR) has been prominent in artificial intelligence applications recently. Among them, modeling the spatial relationship between the 2D images captured by the real camera and the 3D model of the environment (2D and 3D space) is a method to realize the virtual-real registration of augmented reality (AR). Based on this, Yao et al. (2021) proposed a new end-to-end network AE-GAN-Net to verify and evaluate the virtual-real registration performance of AR in the cross-domain image matching results, providing a novel tool for AR virtual-reality registration in outdoor environments.

## 7 Summary and future outlook

Point cloud registration is a crucial task in many areas. This review discusses the point cloud registration technology based on deep learning. Based on the morphology of point clouds, the current methods are categorized into two types: complete overlapping point cloud registration and partial overlapping point cloud registration. For each type, a comprehensive overview and evaluation have been presented. How to further improve the performance of the method, and speed up the processing speed of the network to adapt to the real data on a larger scale is analyzed and summarized from both software and hardware aspects. After that, the practical applications of deep learning point cloud registration in various fields are discussed, and some ideas about the possible development in the future are put forward. At present, the point cloud registration method of deep learning has made great progress, but it still needs to improve. At the end of this article, the future research direction and challenges are prospected.

(1) Influence of point cloud density, scene noise and low overlap on the robustness of the registration methods. The point cloud data obtained in natural environment often has much noise, uneven distribution, and low overlapping. In the future, how to further strengthen the robustness of the method in practical situation s is one of the future research focuses. (2) Real-time processing of massive point cloud data in large-scale situations. In order to apply point cloud registration to actual medical image guidance, intelligent vehicles, and other fields, efficient real-time performance is needed. Due to the complex network structure, it is often difficult for the proposed methods to process massive point cloud data quickly. Therefore, while ensuring the effectiveness of the method, improving the real-time performance of the method is also the focus of future re-search. (3) The registration based on deep learning can be supervised or unsupervised. Most of the existing deep learning point cloud registration methods are supervised and must be based on a large number of training samples. However, the unsupervised method can avoid the calculation cost caused by the need for massive annotated data in the training process. Still, the unsupervised method generally uses the loss function of similarity measurement to train the network, which makes the selection of loss function become extremely important. To solve the above problems, self-monitoring may be a good solution. This method does not require pre-annotated point cloud data for training, but carries out self-annotated training through the inherent internal relationship between input data. This is of great significance to the real-time performance of the method. This is also one of the research hotspots in the future. (4) Non-rigid point cloud registration method. Point clouds can be divided into rigid point cloud registration and non-rigid point cloud registration for different estimation categories. Non-rigid registration is essential for human point cloud data, joints, deformation, etc. Compared with rigid registration, non-rigid point cloud registration needs to consider deformation in addition to the above challenges, which leads to larger solution space. This is also a big challenge for researchers. The current mainstream methods can be divided into two categories: displacement-based and flow-based models. However, only a few researches on non-rigid point cloud registration are based on deep learning. This is undoubtedly one of the research hotspots in the future.

## 8 Conclusion

In conclusion, this review has provided a comprehensive analysis of the current state of point cloud registration technology based on deep learning. It has categorized methods into complete and partial overlapping point cloud registration, highlighted the need for performance improvement, and addressed challenges from both software and hardware perspectives. The practical applications of deep learning in various fields have been explored, offering valuable insights for its future development. The identified research directions include enhancing robustness in noisy and low-overlapping environments, improving real-time processing capabilities, exploring unsupervised learning methods, and advancing non-rigid point cloud registration. These challenges represent exciting areas of future research, underscoring the evolving landscape of deep learning in point cloud registration.

## Author contributions

LC: Funding acquisition, Resources, Supervision, Writing—review & editing. CF: Resources, Supervision, Visualization, Writing—original draft, Writing—review & editing. YM: Data curation, Formal analysis, Funding acquisition, Project administration, Writing—review & editing. YZ: Investigation, Software, Supervision, Writing—review & editing. CW: Methodology, Supervision, Validation, Visualization, Writing—review & editing.
